# Sequential formation of two branched intermediates during protein splicing of class three inteins

**DOI:** 10.1007/s00792-016-0876-0

**Published:** 2016-10-04

**Authors:** Kazuo Tori, Francine Perler

**Affiliations:** 1 New England Biolabs, Inc., Ipswich, MA 01938 USA; 2 Takara Bio USA, Inc., 1290 Terra Bella Ave., Mountain View, CA 94043 USA; 3 Perls of Wisdom Biotech Consulting, Brookline, MA 02446 USA; 4 Department of Molecular Biophysics and Biochemistry, Yale University, New Haven, CT 06520 USA

**Keywords:** Intein, Protein splicing, Branched intermediate, Enzyme mechanism, Cysteine

## Abstract

Inteins are the protein equivalent of introns. They are seamlessly removed during post-translational maturation of their host protein (extein). Inteins from extremophiles played a key role in understanding intein-mediated protein splicing. There are currently three classes of inteins defined by catalytic mechanism and sequence signatures. This study demonstrates splicing of three class 3 mini-inteins: *Burkholderia vietnamiensis G4* Bvi IcmO intein*, Mycobacterium smegmatis* MC2 155 Msm DnaB-1 intein and *Mycobacterium leprae* strain TN Mle DnaB intein. *B. vietnamiensis* has a broad ecological range and remediates trichloroethene. *M. smegmatis* is a biofilm forming soil bacteria. Although other intein classes have only a single branched intermediate at the C-terminal splice junction, the class 3 intein reaction pathway includes two branched intermediates. The class 3 specific branched intermediate is formed by an internal cysteine, while the C-terminal branch intermediate is at a serine or threonine in all class 3 inteins except the Bvi IcmO intein, where it is a cysteine. This latter cysteine was unable to compensate for mutation of the class 3-specific internal catalytic cysteine despite the Bvi IcmO intein having an N-terminal splice junction naturally tuned for a cysteine nucleophile, demonstrating the mandatory order of branch intermediates in class 3 inteins.

## Introduction

Inteins are protein splicing elements that are removed from precursor proteins by a self-catalytic mechanism. Inteins from extremophiles were key to defining all three currently known mechanisms of intein-mediated protein splicing (Brace et al. 2010; Eryilmaz et al. 2014; Mills et al. 2014; Southworth et al. 2000; Tori et al. 2010; Volkmann and Mootz 2013; Xu et al. 1994; Xu and Perler 1996). Over 500 intein genes have been identified in numerous archaea, eubacteria, single cell eukaryotes and viruses (Perler 2002), and the numbers are growing rapidly with the explosion of archaeal and bacterial genome sequences. Inteins disrupt the function of their host protein (termed an extein) and thus protein splicing is required for survival if the intein is present in an essential protein (Dalgaard et al. 1997; Novikova et al. 2014; Perler 2002). The intein and extein are translated as a single, fused precursor protein. During post-translational maturation, the intein removes itself from this precursor while joining the flanking extein fragments with a native peptide bond. No external enzyme or cofactor is required. The majority of inteins are bifunctional enzymes that have a homing endonuclease domain as well as a protein splicing domain. The homing endonuclease is responsible for lateral transmission of intein genes, making them parasitic mobile genetic elements (Barzel et al. 2011; Novikova et al. 2014). Mini-inteins do not have an endonuclease domain, but retain the core protein splicing domain. Modern day mini-inteins are thought to be the descendents of inteins that lost their homing endonuclease domain (Barzel et al. 2011; Novikova et al. 2014).

Very few inteins have been characterized biochemically or even shown to be functional (Perler 2002). In this study we characterized the activity of three mini-inteins: the *Burkholderia vietnamiensis G4* Bvi IcmO intein (Nordberg et al. 2014; Perler 2002), the *Mycobacterium smegmatis* MC2 155 Msm DnaB-1 intein (Mohan et al. 2015; Perler 2002), and the *Mycobacterium leprae* TN Mle DnaB intein (Eiglmeier et al. 1993; Perler 2002). DnaB is a replicative helicase in bacteria. The function of IcmO has yet to be established. The Bvi IcmO gene is present on a *B. vietnamiensis* plasmid, not the main chromosome (Nordberg et al. 2014). *B. vietnamiensis G4* has a broad ecological range, fixes nitrogen, remediates trichloroethene and is found in the lungs of Cystic Fibrosis patients (Nordberg et al. 2014). *M. smegmatis* is a soil bacteria that forms biofilms and *M. leprae* is a slow growing human pathogen.

**Fig. 1:**
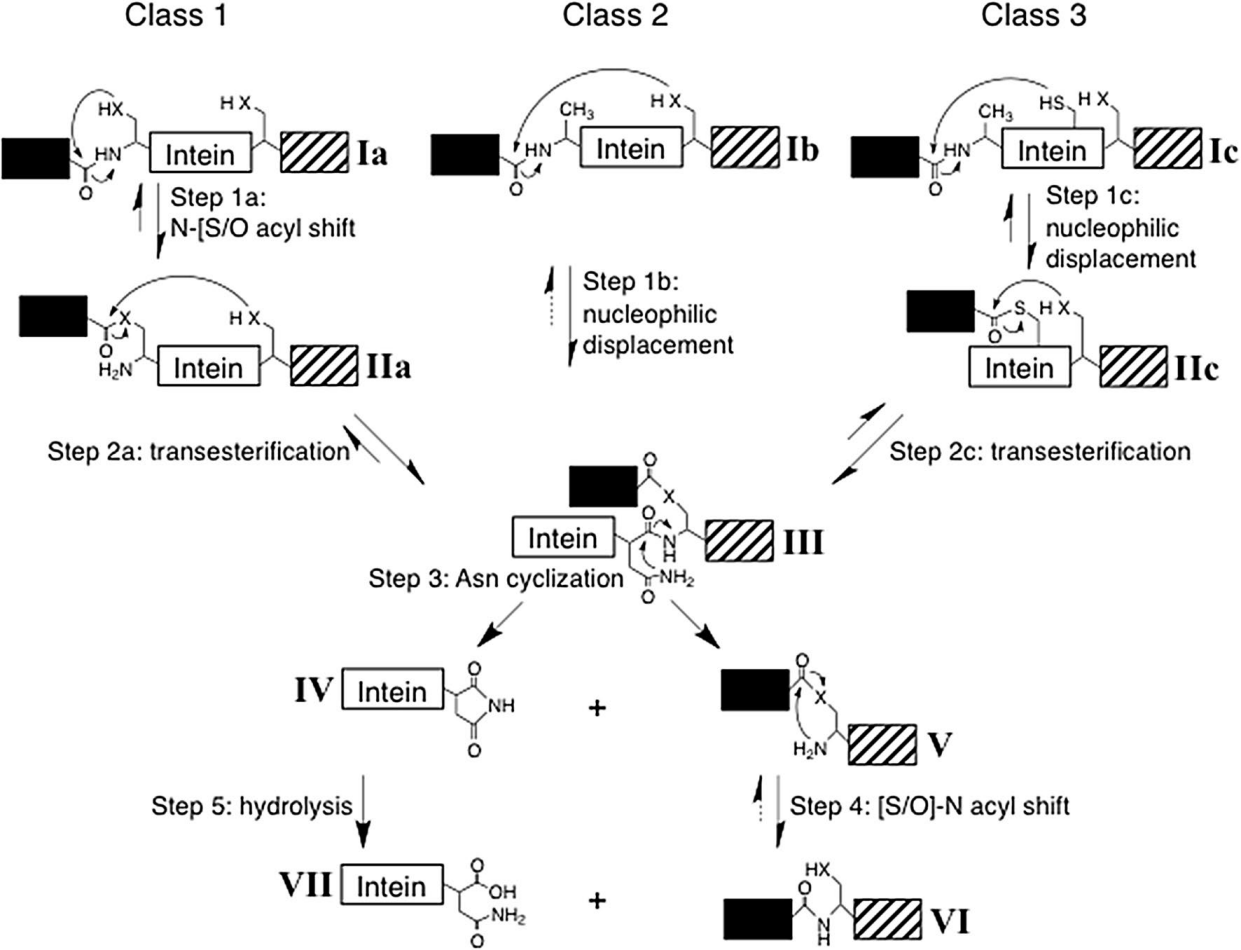
Intein-mediated protein splicing mechanisms. The majority of inteins follow the class 1 intein-mediated protein splicing mechanism, which consists of four coordinated nucleophilic displacements and requires Ser^1^, Thr^1^ or Cys^1^ as the intein N-terminal residue. Step 1a results in a linear (thio)ester intermediate and step 2a results in BI^G^ with Cys^+1^, Ser^+1^ or Thr^+1^ as the branch point. Class 2 and 3 inteins do not require an intein N-terminal nucleophile. Class 2 inteins directly form BI^G^ when the +1 residue attacks the N-terminal splice junction peptide bond. Class 3 inteins use a conserved Cys at Block F position 4 (Cys^F:4^) to initiate protein splicing resulting in formation of the class-specific BI^F^. Once BI^G^ is formed, the remaining reactions are the same for all inteins. The acyl shift in step 4 is rapid and spontaneous. Step 5 is also spontaneous, but is often slow. *Solid arrows* represent steps that have been experimentally verified while *dashed arrows* represent theoretical steps. Note that steps 1 and 2 are reversible; the forward reactions are driven by kinetic rates, equilibrium positions toward the forward reaction, and substrate/intermediate elimination as the protein moves toward the final products, among other factors. Intein residues and flanking extein residues that assist these reactions are not shown, nor are tetrahedral intermediates. ‘X’ represents the sulfur or oxygen atom in the side chain of Cys, Ser or Thr

Inteins are currently divided into three classes based on differences in their protein splicing mechanism (Fig. 1) and conserved signature sequences (Tori et al. 2010). All inteins have at least four conserved motifs (Blocks A, B, F and G) in the splicing domain (Perler 2002; Perler et al. 1997; Pietrokovski 1994, 1998). Amino acids (aa) within these conserved motifs are numbered using the block designation and the position within the block, separated by a colon (Perler 2002; Tori et al. 2010). For example, the fourth amino acid in Block F is referred to as F:4. Several amino acids in these conserved motifs are present at or near the intein active site where they directly act as nucleophiles and facilitating residues for catalysis or they assist in proper packing of the intein active site to align catalytic residues (Brace et al. 2010; Eryilmaz et al. 2014; Kawasaki et al. 1997; Mills et al. 2014; Romanelli et al. 2004; Southworth et al. 2000; Tori et al. 2010; Volkmann and Mootz 2013; Xu et al. 1994; Xu and Perler 1996). Catalytically important residues common to all classes of inteins include His^B:10^ (essential for N-terminal splice junction reactions), the intein penultimate His^G:6^ (assists in C-terminal splice junction reactions), the intein C-terminal Asn^G:7^ (responsible for C-terminal splice junction cleavage during resolution of the Block G branched intermediate, BI^G^) and Ser^+1^, Cys^+1^ or Thr^+1^ [the G:8 residue, responsible for BI^G^ formation and conversion of a (thio)ester bond to an amide peptide bond between the ligated exteins]. Each intein has a larger set of facilitating residues that are tuned to its specific set of nucleophiles (Eryilmaz et al. 2014; Kawasaki et al. 1997; Mills et al. 2014; Romanelli et al. 2004; Tori et al. 2010; Volkmann and Mootz 2013; Wu et al. 2014; Xu and Perler 1996). Moreover, amino acids in the extein, especially proximal ones, influence protein splicing by directly participating in catalysis or by affecting the architecture of the intein active site (Amitai et al. 2009; Cheriyan et al. 2013, 2014; Chong et al. 1997; Eryilmaz et al. 2014; Iwai et al. 2006; Liu et al. 2014; Muona et al. 2010; Perler et al. 1994, 1997; Southworth et al. 1999; Volkmann and Mootz 2013; Xu et al. 1994; Xu and Perler 1996). Class 3 inteins have an additional signature sequence (Table 1) consisting of a dispersed triplet: Trp^B:12^, Cys^F:4^, and Thr^G:5^ (Tori et al. 2010). Previous studies indicated that mutation of Thr^G:5^ can have a minimal effect, while mutation of Trp^B:12^ more significantly impairs splicing, and mutation of Cys^F:4^ totally blocks splicing (Brace et al. 2010; Tori et al. 2010; Tori and Perler 2011). Another feature of class 3 inteins is Ser^+1^ or Thr^+1^ at the G:8 position instead of Cys^+1^, which dominates this position in other intein classes (Brace et al. 2010; Perler 2002; Southworth et al. 2000; Tori et al. 2010; Tori and Perler 2011). The Bvi IcmO intein is the only currently known class 3 intein with Cys^+1^.

**Table 1:**
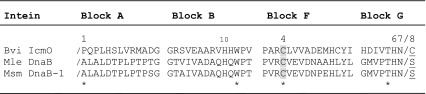
Intein conserved motifs

The sequence of each motif is listed with the intein class 3 signature positions marked by an asterisk. The slash denotes each splice junction. The position number within each block of selected catalytically important amino acids is listed. The class 3 specific BI^F^ branch point (F:4) is shaded gray and the BI^G^ branch point common to all inteins (G:8 or +1) is underlined

Protein splicing requires two or three catalyzed nucleophilic displacement reactions, depending on the intein class, followed by a spontaneous acyl rearrangement that results in a peptide bond between the ligated exteins (Fig. 1). Inteins perform these reactions by functioning as single turnover enzymes. Single turnover enzymes use the same methods as traditional enzymes to achieve catalysis, but do not act on multiple substrates. Although an intein was originally defined as the sequence that is removed from a precursor protein (Perler et al. 1994), when discussing the enzymatic properties of an intein we include the +1 aa (G:8) because it actively participates in splicing. The majority of inteins belong to class 1, which splices in four well-known steps (Eryilmaz et al. 2014; Mills et al. 2014; Perler 2002; Volkmann and Mootz 2013). Class 1 is defined by an intein N-terminal Ser^1^, Thr^1^ or Cys^1^ that forms a linear (thio)ester intermediate (IIa, Fig. 1) prior to forming BI^G^ (III). Class 2 inteins do not have a Cys^1^, Thr^1^ or Ser^1^ (Perler 2002; Southworth et al. 2000). Instead, the +1 aa directly attacks the peptide bond at the N-terminal splice junction (step 1b) to yield a standard BI^G^ (Southworth et al. 2000). Only class 2 inteins can perform step 1b. Class 3 inteins are similar to class 2 inteins since an intein N-terminal Cys^1^, Thr^1^ or Ser^1^ is not required for splicing (Brace et al. 2010; Perler 2002; Tori et al. 2010; Tori and Perler 2011). However, class 3 inteins initiate splicing when the class-specific conserved Cys^F:4^ attacks the peptide bond at the N-terminal splice junction (step 1c) resulting in the formation of the class 3-specific Block F BI (BI^F^, IIc) (Brace et al. 2010; Tori et al. 2010; Tori and Perler 2011). BI^G^ (III) is then formed by a transesterification reaction (step 2c). All inteins follow the same pathway for BI^G^ resolution and formation of the peptide bond between the exteins.

This study examined whether three mini-inteins lacking an N-terminal Ser^1^, Thr^1^ or Cys^1^ are functional and the mechanism by which they splice. For the first time we were able to test whether there is a mandatory order of BI formation in a class 3 intein that naturally has Cys^+1^ and an N-terminal splice junction already tuned for attack by a Cys nucleophile.

## Materials and methods

### Cloning, mutagenesis, and protein expression

All clones were sequenced by the New England Biolabs core facility. The genes for the Msm DnaB-1 and Mle DnaB inteins with flanking DnaB extein sequences and appropriate restriction enzyme sites were synthesized by Invitrogen (Carlsbad, CA, USA). The gene encoding the Bvi IcmO intein with flanking IcmO extein residues was amplified by PCR from *B. vietnamiensis G4* genomic DNA using Phusion DNA polymerase and primers containing *Xho*I and *Spe*I restriction enzyme sites. The Msm DnaB-1 and Mle DnaB intein precursors included native DnaB flanking residues Phe-Gly-Val-Gly-Lys (N-extein) and Ser-Thr-Leu-Gly-Leu (C-extein), while the Bvi IcmO intein precursor included native flanking IcmO residues Ala-Arg-Ser-Leu-Gly-Phe (N-extein) and Cys-Ile-Thr-Phe-Ala (C-extein). The DNAs were digested by *Xho*I and *Spe*I, agarose gel purified and ligated into pMP1 (Southworth et al. 2000; Tori et al. 2010) previously digested with the same enzymes. This resulted in pMSP with the Msm DnaB-1 intein, pMLP with the Mle DnaB intein and pMVP with the Bvi IcmO intein, where the intein was flanked with the *Escherichia coli* maltose-binding protein (M or MBP) and the Δ*Sal* fragment of *Dirofilaria immitis* paramyosin (P).

By convention, amino acids in the intein are numbered beginning with the intein N-terminus and residues in the C-extein are numbered similarly, but contain a plus sign to denote the C-extein. His^B:10^, Trp^B:12^, Cys^F:4^ and Asn^G:7^ are, respectively, His^65^, Trp^67^, Cys^118^ and Asn^139^ in the Msm DnaB-1 intein, His^65^, Trp^67^, Cys^124^ and Asn^145^ in the Mle DnaB intein and His^65^, Trp^67^, Cys^122^ and Asn^142^ in the Bvi IcmO intein (Perler 2002).

All mutations were made using the Phusion site-directed mutagenesis kit (New England Biolabs) with primers that introduced the desired mutation. For protein expression, freshly transformed *E.coli* NEB Turbo cells were grown in LB media containing 100 µg/ml ampicillin at 37 °C until reaching an OD_600_ of ~0.5 and then induced with 0.4 mM IPTG at room temperature, 30 °C and 37 °C for 2 h or at 15 °C overnight. Protein splicing was assessed using soluble lysates after electrophoresis in 4–20 % SDS-PAGE (Invitrogen, Carlsbad, CA, USA). Proteins were detected by either Simply Blue Safe Stain (Invitrogen) or fluorescent Western Blot as described previously (Cheriyan et al. 2013; Southworth et al. 2000; Tori and Perler 2011). Briefly, nitrocellulose filters were concurrently probed with mouse anti-MBP sera and rabbit anti-paramyosin sera, and then developed concurrently with IR-Dye 680 anti-mouse secondary antibody (green) or IR-Dye 800 anti-rabbit secondary antibody (red) (LI-COR, Lincoln, NE, USA).

### Purification and characterization of branched intermediates

BIs of Msm DnaB-1 and Mle DnaB inteins with Asn^G:7^ mutated to alanine in MVP and MLP were purified by affinity chromatography over amylose resin. The pH of an aliquot of each purified BI was changed by addition of sodium phosphate buffer. Samples were incubated overnight at room temperature in the absence or presence of 50 mM DTT. Time zero (*T*_0_) samples were heated at 100 °C for 5 min in SDS-PAGE sample buffer (New England Biolabs) without incubation. An aliquot of purified MLP BI was denatured by adding solid urea to a final concentration of 8 M. The pH of the denatured sample was checked prior to subsequent treatment as above for native samples.

## Results

### Splicing of three mini-inteins in vivo

**Fig. 2:**
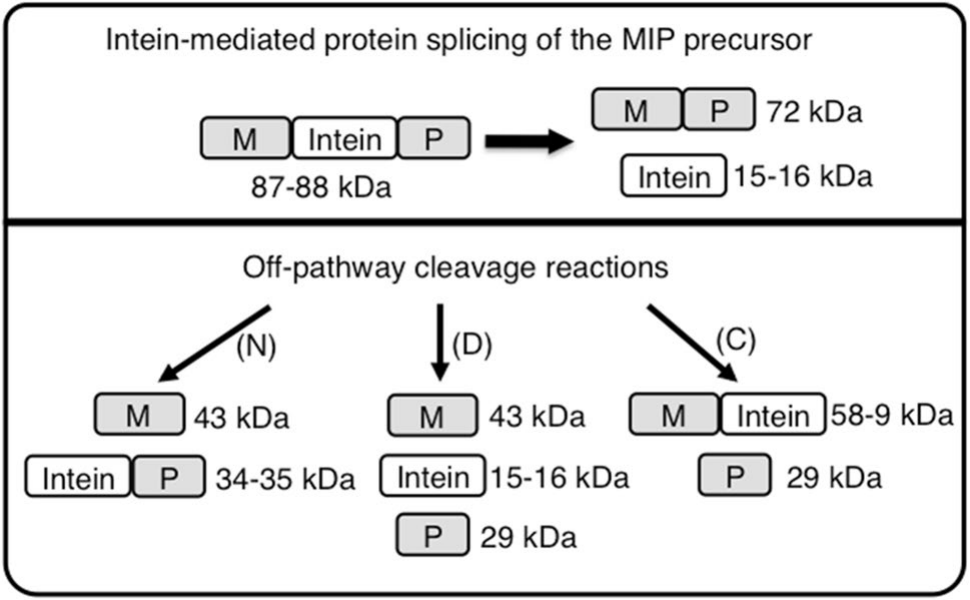
Splicing and cleavage schemes for the MIP precursor with either the Bvi IcmO, Mle DnaB or Msm DnaB-1 inteins. Off-pathway cleavage reactions can occur at the N-terminal splice junction (N), the C-terminal splice junction (C) or both splice junctions (D). Branched intermediate decay yields the same products as direct N-terminal splice junction cleavage (N)

DNAs encoding the Msm DnaB-1 (139 aa) and Mle DnaB (145 aa) mini-inteins along with 5 DnaB extein residues flanking the intein on each side (Perler 2002) were synthesized and cloned in the MIP model precursor system (Xu et al. 1994) between the *E. coli* maltose binding protein (MBP or M) and the *D. immitis* paramyosin Δ Sal fragment (P) generating precursors MSP and MLP, respectively. The coding sequence for the Bvi IcmO intein (142 aa) was amplified by PCR with flanking IcmO extein residues and likewise cloned into MIP generating MVP. Splicing of these model precursors (Fig. 2) results in production of MP (72 kDa) plus free intein (I, 14.7–16.0 kDa). Off-pathway cleavage reactions would result in production of M (43 kDa) and IP (34–35 kDa) after N-terminal cleavage or BI decay, and MI (58–59 kDa) and P (29 kDa) after C-terminal cleavage.

**Fig. 3:**
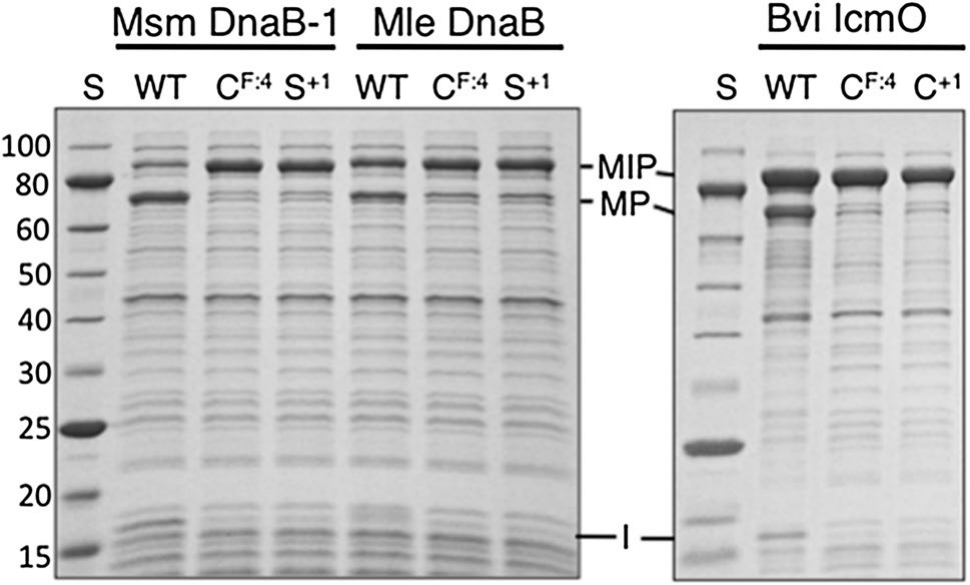
Splicing of wild type and mutant inteins in model precursors. MSP (Mle DnaB intein) and MLP (Msm DnaB-1 intein) were expressed at 15 °C and MVP (Bvi IcmO intein) was expressed at 30 °C. In vivo splicing of soluble proteins was examined after electrophoresis in SDS-PAGE followed by staining with Simply Blue Safe Stain. Residues mutated to alanine are listed above each lane. *Lane S* contains the NEB 10–250 kDa Protein Ladder with molecular masses (kDa) listed on the left. *MIP* precursor with the indicated inteins, *MP* spliced product, *I* excised intein, *WT* wild type intein

Splicing in model systems can vary at different temperatures due to differences in expression rates, precursor folding and aggregation. Therefore, in vivo splicing activity of all three inteins was examined after each precursor was expressed in *E. coli* at 37 °C, 30 °C and room temperature for 2 h or at 15 °C overnight. Msm DnaB-1 and Mle DnaB inteins yielded spliced products only at 15 °C (Fig. 3 and data not shown). MVP spliced poorly with less than half of the MVP precursor converted to spliced product at all temperatures tested (Fig. 3 and data not shown). These results demonstrate that all three mini-inteins are active, although the degree of splicing in these model precursors varied with the intein. Low levels of spliced product generally reflect misfolding of model precursors, especially when the precursor accumulates as a nonreactive component.

### Dissecting the splicing pathway by mutation of conserved amino acids

Although all three inteins have the class 3 signature motif (Table 1), they can potentially splice by either the class 2 or class 3 mechanism. Mutation of Cys^F:4^ can distinguish between these splicing mechanisms because it blocks the first step in the class 3 splicing pathway, but not in the class 2 pathway (Fig. 1). No splicing or cleavage was observed in MLP, MSP or MVP after substitution of Cys^F:4^ with alanine when analyzed by Simply Blue Safe Stained SDS-PAGE or florescent Western Blot (Fig. 3 and data not shown). Only precursor was observed when His^B:10^ or Trp^B:12^ was mutated to alanine in all three inteins, which is consistent with their importance for splicing of class 3 inteins. Nonconservative substitution of Ser^+1^ or Cys^+1^ to alanine yielded unreacted precursor in Simply Blue Safe Stained SDS-PAGE and by florescent Western Blot analysis (Fig. 3 and data not shown). However, splicing was observed after the conservative substitution of Ser^+1^ with Cys in MSP and MLP, while substitution of Cys^+1^ with Ser blocked splicing and yielded only unreacted MVP precursor as analyzed in florescent Western Blots and stained SDS-PAGE (data not shown). These results are consistent with data from many other inteins where cysteine could substitute for a catalytic serine or threonine, but serine could not substitute for a catalytic cysteine; these effects are attributed to differences in pKa and the greater need to activate serine or threonine side chain hydroxyls to increase their nucleophilicity compared to cysteine side chain thiols (Eryilmaz et al. 2014; Mills et al. 2014; Volkmann and Mootz 2013). Such differences in reactivities and overall amino acid size are factors contributing to the tuning of an enzyme to its specific catalytic residues. Taken together, the mutation data indicate that all three mini-inteins follow the class 3 splicing pathway (Fig. 1).

### In vitro analysis of the Msm DnaB-1 and Mle DnaB intein branched intermediates

**Fig. 4:**
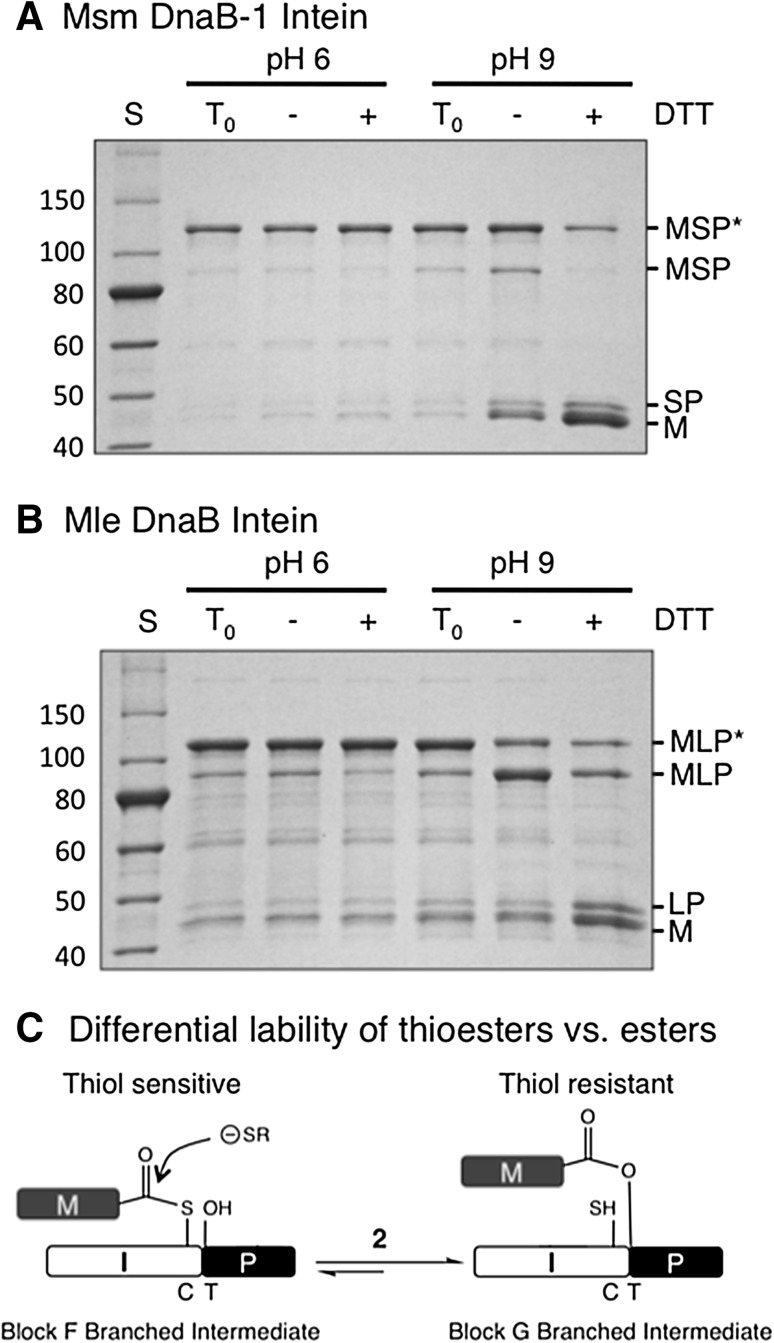
Investigation of MSP and MLP branched intermediates in vitro. MSP (**a**) and MLP (**b**) BIs accumulated in vivo when the intein C-terminal Asn^G:7^ was mutated to alanine. Purified BIs (MSP* or MLP*) were incubated in vitro at pH 6 or 9 in the absence (−) or presence (+) of 50 mM DTT at room temperature. Time zero samples (*T*_0_) were not incubated in vitro. The SDS-PAGE gel was stained with Simply Blue Safe Stain. *Lane S* NEB 10–250 kDa Protein Ladder with molecular masses (kDa) listed on the left of the gels. **c** Step 2 in the splicing pathway of class 3 inteins reversibly converts BI^F^ to BI^G^. Under the conditions of these experiments, thiol reagents can attack a thioester bond, but not an ester bond

Asn^G:7^ to alanine mutations in MSP and MLP resulted in BI accumulation (Fig. 4), which is normally seen as a slowly migrating band in SDS-PAGE compared to the initial precursor. These results are consistent with the role of Asn^G:7^ in branch resolution (step 3, Fig. 1). The nature of the BIs that accumulated in vivo was then examined in vitro. Purified BI samples from MSP and MLP after Asn^G:7^ to alanine substitution were incubated overnight at room temperature at either pH 6 or pH 9 in the presence or absence of 50 mM DTT (Fig. 4). The BIs were stable at pH 6 under all conditions tested. At pH 9, in the presence of DTT the BIs decayed to form M + SP or LP, while in the absence of DTT the BIs partially reverted back to MSP or MLP precursors. Stability was also tested with denatured MLP BI. No change was observed upon incubation of the denatured MLP BI sample overnight at room temperature with 50 mM DTT at either pH (data not shown), indicating that thiol induced BI decay requires a properly folded protein and that the steady state BI molecules do not have detectable amounts of a thiol sensitive bond.

### Characterization of catalytically important cysteines in the Bvi IcmO intein

The Bvi IcmO intein is the only currently identified class 3 intein with Cys^+1^ and thus both BI^F^ and BI^G^ have a thioester linkage. Mutation of the Bvi IcmO intein Asn^G:7^ did not result in BI accumulation in vivo and instead yielded N-terminal splice junction cleavage products (M + VP). This is consistent with previous studies that demonstrated in vivo lability of BI thioester linkages (Brace et al. 2010; Mills et al. 2014; Southworth et al. 2000; Tori et al. 2010; Tori and Perler 2011; Volkmann and Mootz 2013).

**Fig. 5:**
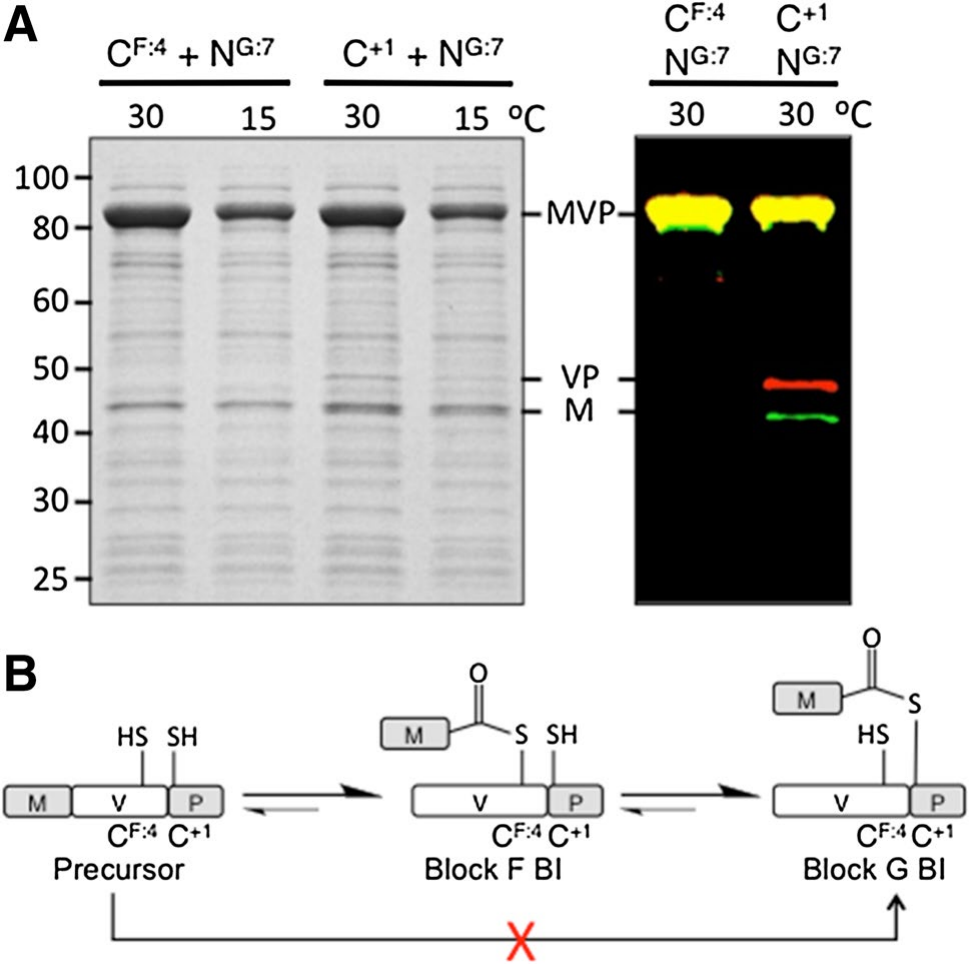
Analysis of alanine substitutions at conserved cysteines in the Bvi IcmO intein. **a** Double mutations were made in the MVP precursor substituting alanine for Asn^G:7^ and either Cys^F:4^ or Cys^+1^. The Asn^G:7^ substitution prevents on-pathway BI^G^ resolution. MVP precursors with the indicated residues mutated to alanine were expressed at 30 °C for 2 h or 15 °C overnight. *Left panel* SDS-PAGE stained with Simply Blue Safe Stain. *Right panel* Western Blots were probed using IR-dye tagged secondary antibodies with M shown in *green* and P shown in *red*. *Yellow* bands indicate an equal reaction with both secondary antibodies. **b** The forward and reverse reaction scheme for MVP precursor to BI^G^. The *red* ‘X’ indicates that this reaction was not detected

The Bvi IcmO intein provides a unique opportunity to examine any potential competition between the catalytic cysteines at F:4 and G:8 (+1), and to determine if Cys^+1^ can directly attack an N-terminal splice junction that is already tuned for cleavage by a cysteine. Single alanine substitutions of Cys^F:4^ or Cys^+1^ yielded only unreacted precursor in both stained gels and fluorescent Western Blots (Fig. 3 and data not shown). However, when alanine substitutions of each cysteine were combined with alanine substitutions of Asn^G:7^, the Cys^+1^ double mutant produced N-terminal cleavage products while the Cys^F:4^ double mutant did not, as assayed in stained SDS-PAGE and confirmed in the more sensitive fluorescent Western Blot (Fig. 5). Further experimentation is necessary to determine why cleavage products were not observed with the single MVP Cys^+1^ mutant, especially structural studies of wild type and mutant inteins in MVP. It is possible that the Asn^G:7^ mutation opens up the active site for in vivo cleavage of the thioester linkage in BI^F^.

## Discussion

The Msm DnaB-1, Mle DnaB and Bvi IcmO inteins should be class 3 inteins based on their sequence signatures (Tori et al. 2010) and phylogenetic analysis (Tori and Perler 2011). This study proved that all three inteins splice using the class 3 mechanism as demonstrated by (1) the absolute requirement of Cys^F:4^ for splicing and N-terminal splice junction cleavage, (2) mutation of the class 3 specific signature residue Trp^B:12^ blocked splicing, and (3) the inferred presence of BI^F^ by thiol induced decay of purified BIs from MSP and MLP. It is interesting to note that C-terminal cleavage by Asn^G:7^ in all three inteins appears to be strongly coupled to earlier steps in the splicing reaction as observed previously in some other inteins (Martin et al. 2001; Mills et al. 2014; Volkmann and Mootz 2013), since His^B:10^, Trp^B:12^, Cys^F:4^, Ser^+1^ and Cys^+1^ mutations did not yield C-terminal splice junction cleavage products.

It is difficult to experimentally distinguish between BI^G^ (III, Fig. 1) and BI^F^ (IIc) because they have the same mobility in SDS-PAGE, the same mass and have not been detected by Mass Spectrometry. Instead, indirect approaches have been employed to identify BIs, especially the difference in lability of BI thioester vs. ester linkages in vivo and in vitro. Thioester-linked BIs rarely accumulate in *E. coli*, while ester-linked BIs are often detected (Brace et al. 2010; Mills et al. 2014; Southworth et al. 2000; Tori et al. 2010; Tori and Perler 2011; Volkmann and Mootz 2013; Xu et al. 1994; Xu and Perler 1996). The in vivo instability of thioester linked BIs was observed in this study since only products of BI thiolysis accumulated in MVP (both BIs have a thioester linkage) after BI^G^ resolution was prevented by mutation of Asn^G:7^. Based on (thio)ester stability, the MSP and MLP BIs that accumulated in vivo after alanine substitution of Asn^G:7^ are most likely the ester-linked BI^G^ rather than the labile thioester linked BI^F^ (Fig. 4c). These ester-linked BIs should be stable under the mild conditions used for in vitro thiolysis experiments. However, at pH 9 both the MSP and MLP BIs decayed during DTT treatment. At first glance, these results seem inconsistent because the in vivo data suggest accumulation of the ester linked BI^G^, while the in vitro data suggest the presence of the thioester containing BI^F^. The reversibility of step 2c (Figs. 1, 5b), explains this conundrum (Brace et al. 2010; Tori et al. 2010; Tori and Perler 2011). The reverse reaction was directly observed when MSP and MLP BIs converted back to linear precursors (Ic) at pH 9 in the absence of DTT (Fig. 4). If DTT is present during the reverse reaction, BI^F^ is eliminated by DTT and purified BI^G^ continually converts to BI^F^ to maintain the equilibrium between the two BIs, eventually resulting in the complete disappearance of both BIs due to thiolysis of BI^F^. The observation that DTT was unable to cleave denatured MLP BI confirms the hypothesis that BI^G^ and not BI^F^ accumulates in vivo because direct cleavage of BI^G^ by thiols would not occur under the experimental conditions (Fig. 4c) and denaturation only prevents forward or reverse reactions while leaving any (thio)ester bond intact.

The Bvi IcmO intein provided a sensitive means of analyzing the mandatory order of BI formation in class 3 inteins. It is possible that Cys^+1^ can compete with Cys^F:4^ for attack on the peptide bond at the N-terminal splice junction because this residue normally attacks the N-terminal splice junction in other intein classes during steps 2a or 1b (Eryilmaz et al. 2014; Mills et al. 2014; Southworth et al. 2000; Volkmann and Mootz 2013). Although previous studies with other class 3 inteins demonstrated that Ser^+1^ cannot initiate the splicing reaction, they all required Ser^+1^ to attack an N-terminal splice junction that was primed for attack by a cysteine nucleophile and thus may not work because of mechanistic differences caused by the different nucleophiles (Brace et al. 2010; Tori et al. 2010; Tori and Perler 2011). The Bvi IcmO intein allows testing of a naturally occurring Cys^+1^ in a class 3 intein without the problems associated with changing the nucleophile. Cys^+1^ did not generate any BI or N-terminal splice junction cleavage products in the sensitive fluorescent Western Blot assay of the MVP Cys^F:4^ plus Asn^G:7^ double mutant under conditions where cleavage products were produced by Cys^F:4^ in the MVP Cys^+1^ plus Asn^G:7^ double mutant. It is unlikely that undetected BI^G^ formed or decayed in this experiment because the IR dyes used can detect picogram amounts of protein and microgram amounts of MVP precursor were queried. These results demonstrate that (1) the presence of a natural Cys^+1^ cannot substitute for the loss of Cys^F:4^ by mutation and (2) the order of BI formation in class 3 inteins must be BI^F^ followed by BI^G^ (Fig. 5b). This reaction order is likely maintained by local changes at the intein active site after formation of BI^F^ that are required to properly align or activate Cys^+1^ for the next catalytic step.

In summary, this study provides experimental evidence that all three class 3 mini-inteins are active. We conclude that there is no competition between the two catalytic cysteines for attack on the N-terminal splice junction and that the Bvi IcmO intein is unable to splice by the class 2 mechanism. The Bvi IcmO intein expands the repertoire of potential insertion sites for class 3 inteins in target proteins to include Cys for the numerous in vivo and in vitro applications based on intein technology (Aranko et al. 2014; Topilina and Mills 2014; Wood and Camarero 2014). Inteins continue to prove to be intriguing and robust single turnover enzymes.

## Abbreviations

aa: Amino acid
BI: Branched intermediate
BI^F^: Branched intermediate with Cys^F:4^ as the branch point
BI^G^: Branched intermediate with the +1 (G8) amino acid as the branch point
M or MBP: Maltose binding protein
P: Paramyosin Δ Sal fragment
I: Intein
MIP, MSP, MLP and MVP: Model precursors with MBP and P flanking either any intein, the Msm DnaB-1 intein, the Mle DnaB intein or the Bvi IcmO intein, respectively
